# Development of a Chimeric Porcine Reproductive and Respiratory Syndrome Virus (PRRSV)-2 Vaccine Candidate Expressing Hypo-Glycosylated Glycoprotein-5 Ectodomain of Korean Lineage-1 Strain

**DOI:** 10.3390/vetsci9040165

**Published:** 2022-03-29

**Authors:** Hwi-Yeon Choi, Min-Sik Kim, Yeong-Lim Kang, Jong-Chul Choi, In-Yeong Choi, Sung-Won Jung, Ji-Yun Jeong, Min-Chul Kim, Seong-Soo Hwang, Sang-Won Lee, Seung-Yong Park, Chang-Seon Song, In-Soo Choi, Joong-Bok Lee

**Affiliations:** 1 Laboratory of Infectious Diseases, College of Veterinary Medicine, Konkuk University, 120 Neungdong-ro, Gwangjin-gu, Seoul 05029, Korea; 2839gnl@naver.com (H.-Y.C.); kimminsik9206@hanmail.net (M.-S.K.); vinlovehole@naver.com (Y.-L.K.); mfilia@naver.com (J.-C.C.); joealpha@naver.com (I.-Y.C.); tjddnjs0204@naver.com (S.-W.J.); wldbs3290@konkuk.ac.kr (J.-Y.J.); odssey@konkuk.ac.kr (S.-W.L.); paseyo@konkuk.ac.kr (S.-Y.P.); songcs@konkuk.ac.kr (C.-S.S.); ischoi@konkuk.ac.kr (I.-S.C.); 2 Careside Co. Ltd., Woolim Lions Valley A-B210, #146-8, Sangdaewon-dong, Jungwon-gu, Seongnam 13209, Gyeonggi-do, Korea; rnd4@careside.co.kr; 3 Samhwa Breedings Agri. Inc., 435, Sinjin-ri, Gwangcheon-eup, Hongseong-gun 35090, Chungcheongnam-do, Korea; manofh@naver.com; 4 KU Research Center for Zoonosis, 120 Neungdong-ro, Gwangjin-gu, Seoul 05029, Korea

**Keywords:** PRRSV, chimera, vaccine, Korea, GP5, neutralizing antibodies

## Abstract

Vaccination is a practical method to provide protection against porcine reproductive and respiratory syndrome virus (PRRSV), but current PRRSV vaccines show limited efficacy against divergent field strains. Lineage 1 PRRSV includes virulent strains such as NADC30 and MN184 and now has become one of the most prevalent viruses in Korea. Accordingly, there is an urgent need to develop a new vaccine for Korean lineage-1 strains. In this study, a vaccine candidate against Korean lineage-1 PRRSV, vCSL1-GP5-N33D, was developed by reverse genetics technology. vCSL1-GP5-N33D was designed as a hypo-glycosylated chimeric virus containing the glycoprotein 5 ectodomain region of the Korean lineage-1 wild-type strain. An inactivated vaccine of vCSL1-GP5-N33D was applied to a PRRS-endemic farm and elicited high serum virus neutralization (SVN) antibody titers. The vaccinated group induced SVN antibody titers of 4.40 (log_2_) ± 2.46, which were approximately 2-fold higher than those of the negative control at 8-weeks post-vaccination. Moreover, 60% of pigs in the vaccinated group displayed SVN antibody titers of ≥5 (log_2_), while none of the pigs in the negative control exhibited SVN antibody titers of ≥5 (log_2_). The overall results of the animal experiment suggest that the vCSL1-GP5-N33D inactivated vaccine is a promising vaccine candidate.

## 1. Introduction

Porcine reproductive and respiratory syndrome virus (PRRSV), the etiological agent of PRRS, is a pathogen that adversely impacts global swine production in the economic aspects [1]. PRRSV belongs to the family *Arteriviridae*, order *Nidovirales*, and has a linear positive-sense single-stranded RNA genome of 15 kb [2]. The PRRSV genome includes 10 open reading frames (ORFs). ORFs 1a and 1b encode 16 nonstructural proteins essential for viral replication, and ORFs 2a, 2b, 3-7, and 5a encode structural proteins, including glycoprotein (GP) 2a, E, GP3, GP4, GP5, M, N, and ORF5a proteins, respectively [3].

Major clinical features of PRRS are respiratory disease in growing pigs and reproductive failure in breeding animals [4]. Vaccination is regarded as a practical way to confer protection against PRRSV and prevent clinical signs [5,6]. Both modified live virus (MLV) vaccines and inactivated vaccines are commercially available. The MLV vaccines provide efficacious homologous protection but have safety issues such as reversion to virulence and provide incomplete cross-protection [3,7]. The inactivated vaccines are safe but are known to be less effective even against homologous challenges [4,6]. All vaccines have advantages and disadvantages, although there is broad agreement that current PRRSV vaccines provide limited efficacy against heterologous field viruses, which is closely related to the variable characteristic of the PRRSV genome [6].

PRRSV is divided into two species; *Betaarterivirus suid* 1 (i.e., PRRSV-1) and *Betaarterivirus suid* 2 (i.e., PRRSV-2) [8]. PRRSV-1 and PRRSV-2 share 60% nucleotide sequence identity and high levels of genetic variation (>20%) exist within each species [9]. Accordingly, PRRSV-1 and PRRSV-2 are further divided into four subtypes and nine lineages, respectively, based on the phylogenetic analysis of ORF5 nucleotide sequences [10]. Among the various strains, lineage 1 PRRSV-2 is currently the most prevalent strain in the USA and has become one of the major pathogens in Korea as well [9,11]. In Korea, lineage 5 was dominant from 2014 (46.7%) to 2019 (31.1%), but the lineage 1 population increased to the second-largest population from 2014 (1.8%) to 2019 (29.6%) [12]. Lineage 1 contains virulent strains such as NADC30 and MN184 and many field isolates of lineage 1 were also reported to be virulent [9,10,13]. In the USA, Prevacent PRRS (Elanco, Indianapolis, IN, USA), a PRRSV MLV vaccine derived from a lineage-1 strain, has been commercially available since 2018 [4]. In Korea, however, there are no lineage-1 vaccines available at present. Therefore, a new vaccine against Korean lineage-1 strains is needed for the control of the disease.

PRRSV vaccines are traditionally produced by successive passage in cell lines such as MARC-145 cells [14]. Therefore, it takes years to generate a vaccine candidate, and vaccines for MARC-145-unadpative strains cannot be produced. In this study, reverse genetics technology was utilized to develop a vaccine against a MARC-145-unadpative lineage-1 PRRSV isolated in Korea. The vaccine candidate was designed as a chimeric virus containing the hypo-glycosylated GP5 ectodomain region of the target virus. An inactivated vaccine using this mutant virus was administered to a PRRS-positive herd, and vaccine performance was measured by clinical signs and antibody production.

## 2. Materials and Methods

### 2.1. Cells and Viruses

MARC-145 cells were grown in Dulbecco’s modified Eagle’s medium (DMEM) supplemented with 10% fetal bovine serum (FBS) and 1% antibiotic/antimycotic solution [15]. Porcine alveolar macrophages (PAMs) were collected from lungs of PRRS-free piglets and maintained in RPMI 1640 medium supplemented with 10% FBS and 1% antibiotic/antimycotic solution. All cultures were maintained at 37 °C in a humidified incubator containing 5% CO_2_.

A PRRSV-2 isolate CSNA11 (Genbank accession no: OM777142) and KU-PRRSV-2020-002 (Genbank accession no: OM037453) were isolated from a Korean farm and used in this study. CSNA11 and KU-PRRSV-2020-002 belonged to lineages 5 and 1 in the phylogenetic tree, respectively.

### 2.2. Generation of Mutant Viruses

**Figure 1:**
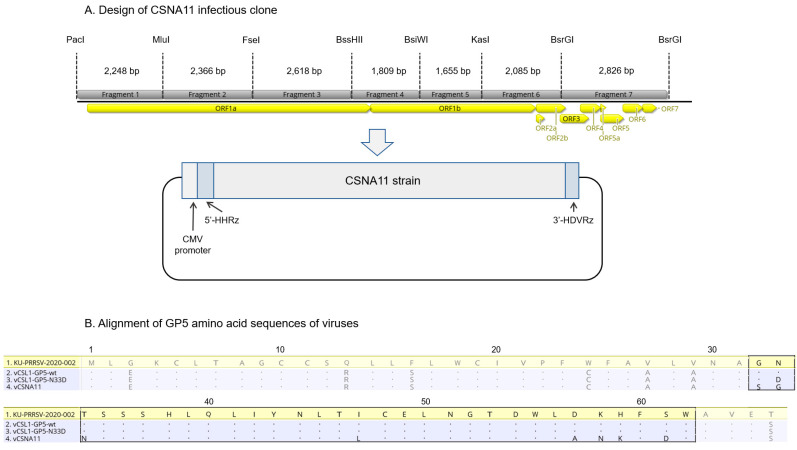
Design of CSNA11 infectious clone and alignment of glycoprotein (GP) 5 amino acid sequences of viruses. (**A**) Schematic diagram of CSNA11 infectious clone. The restriction enzyme sites used for assembling the infectious clone and the length of each fragment are indicated above the genomic construct. CMV: cytomegalovirus; HHRz: hammerhead ribozyme; HDVRz: hepatitis delta virus ribozyme. (**B**) Alignment of amino acid sequences of the GP5 N-terminal domain of viruses. Amino acids identical to those of the KU-PRRSV-2020-002 strain are represented as dots, and the ectodomain region (31 amino acids, 93 bp) is indicated by the black box.

A DNA-launched infectious clone of PRRSV, pCSNA11, was constructed by assembling seven fragments that cover the entire genome of the CSNA11 strain and used as the backbone for cloning in this study (Figure 1A). CSNA11 strain grew to high titers in MARC-145 cells, so infectious clone-derived viruses were expected to have good replicative fitness.

A chimeric cDNA clone, pCSL1-GP5-wt, was designed in silico using Geneious Prime (Biomatters, Auckland, New Zealand) to replace the GP5 ectodomain region of CSNA11 with that of KU-PRRSV-2020-002 strain (Figure 1B). The fragment containing the chimeric GP5 sequence was synthesized de novo (GenScript, Hong Kong, China) and cloned into the full-length cDNA clone to replace the backbone sequence. The chimeric cDNA clone (pCSL1-GP5-wt) was then used to generate a mutant plasmid, pCSL1-GP5-N33D, carrying a GP5 glycosylation site mutation by the QuikChange II XL site-directed mutagenesis kit (Agilent, Palo Alto, CA, USA) according to the manufacturer’s instructions (Figure 1B).

Recombinant infectious clones were transfected into MARC-145 cells using the ViaFect transfection reagent (Promega, Madison, WA, USA) according to the manufacturer’s protocol. At 96 h after transfection, culture supernatants were collected and passaged onto MARC-145 cells.

### 2.3. Indirect Immunofluorescence Assay (IFA)

IFA was performed at 48 h postinfection (p.i.) to detect the viral antigens and confirm the rescue of viruses. Infected MARC-145 cells were fixed and stained with mouse monoclonal antibody to PRRSV-2 nucleocapsid (N) protein (Median diagnostics, Chuncheon, Korea), which were then incubated with anti-mouse Alexa Fluor 488-labeled secondary antibody (Thermo Fisher Scientific, Cleveland, OH, USA).

### 2.4. Growth Properties and Kinetics of Rescued Viruses

For comparison of the growth properties of rescued viruses, viral titers were determined in MARC-145 cells and PAMs by titration and expressed as 50% tissue culture infectious dose (TCID_50_)/mL.

Growth kinetics of rescued viruses were compared in MARC-145 cells. Confluent monolayers of MARC-145 cells in 96-well plates were infected with the rescued viruses at a multiplicity of infection (MOI) of 0.1. After 1 h of viral absorption at 37 °C, cells were washed twice and incubated in a fresh medium. At 12, 24, 48, 72, and 96 h p.i., culture supernatants were collected and stored at −70 °C until use. Viral titers were measured by titration in MARC-145 cells and expressed as TCID_50_/mL.

### 2.5. Preparation of Inactivated Vaccine

The inactivated vaccine was produced by diluting the mutant virus, vCSL1-GP5-N33D, to 2 × 10^7^ TCID_50_/mL and inactivating the virus with binary ethylamine (BEI) as previously described [16,17]. Briefly, a BEI stock solution of 0.1 M was prepared by cyclization of 0.1 M 2-bromoethylamine hydrobromide in 0.175 M NaOH for 1 h at 37 °C. Then, BEI was added to the diluted virus (2 × 10^7.0^ TCID_50_/mL) in a final concentration of 1 mM. After incubation with agitation at 37 °C for 24 h, BEI inactivation was terminated by adding a sodium thiosulfate stock solution of 1 M to a final concentration of 0.1 mM. The BEI-treated virus was passaged onto the MARC-145 cells to verify that the virus was completely inactivated and not infective. The inactivated vaccine of 10^7.0^ TCID_50_/1 mL/dose was manufactured by mixing the viral antigen with Montanide IMS1313 VG adjuvant (SEPPIC, Castres, France) in a 1:1 ratio.

### 2.6. Animal Experiment

A farrow-to-finisher farm (550 sows) endemically infected with a wild-type PRRSV-2 strain, KU-PRRSV-2020-002, was selected to evaluate the effect of the inactivated vaccine of vCSL1-GP5-N33D. This farm of crossbred (large white-landrace-duroc triple cross) swine was under a routine vaccination program for porcine circovirus-2 and Mycoplasma hyopneumoniae.

A total of 15 three-week-old castrated piglets were randomly divided into two groups by the random number generator (SPSS Inc., Chicago, IL, USA). Groups A and B consisted of 10 and 5 pigs each, the sample size of which was determined by the resource equation method [18]. The piglets were housed in separate pens and acclimatized for 4 days before the initiation of the experiments. The investigators were blinded to the group allocation throughout the experiments.

Group A was immunized intramuscularly (IM) at the left side of the neck (needle 23G, 1″ long) with the inactivated vaccine of vCSL1-GP5-N33D (10^7.0^ TCID_50_/1 mL/dose). Group B was injected IM (same condition as group A) with phosphate-buffered saline (PBS) and acted as a negative control. Clinical symptoms were monitored daily during the experiment, and body weights were recorded at 8 weeks post-vaccination (wpv). Enzyme-linked immunosorbent assay (ELISA) titers were measured on sera of 0, 4, 6, and 8 wpv using the IDEXX PRRS X3 Ab Test (IDEXX Laboratories, Inc., Columbus, OH, USA) following the manufacturer’s instructions. Sera collected at 0 and 8 wpv were also assessed for the amount of PRRSV-specific neutralizing antibodies using the serum virus neutralization (SVN) test.

There were no inclusion or exclusion criteria, and no animals, experimental units, or data points were excluded from this study. All experimental protocols were approved by the Institutional Animal Care and Use Committee of Konkuk University (No. KU21117).

### 2.7. SVN Test

An SVN test was performed in MARC-145 cells using the mutant virus, vCSL1-GP5-N33D. Heat-inactivated serum samples were serially diluted two-fold in the medium. Each diluted sample was then mixed with an equal volume of virus containing 4 × 10^3.0^ TCID_50_/1 mL. The mixtures were incubated at 37 °C for 1 h and inoculated onto confluent MARC-145 cell monolayers in 96-well plates. The cells were monitored daily for cytopathic effect (CPE), and antibody titers were measured by CPE after 5 days of incubation.

### 2.8. Statistical Analysis

Researchers were blinded for group allocation during the data analysis process. Due to the small sample size of groups A (*n* = 10) and B (*n* = 5), nonparametric tests including the Kruskal–Wallis test and Fisher’s exact test were used to analyze the differences between the groups. The data were presented as mean ± standard deviation (SD), and a *p*-value of ≤0.05 was considered to be statistically significant. The statistical analysis was performed using IBM SPSS Statistics for Windows, Version 25.0 (IBM Corp., Armonk, NY, USA), and graphs were drawn by GraphPad Prism for Windows, Version 6.01 (GraphPad Software, San Diego, CA, USA).

## 3. Results

### 3.1. Generation of Mutant Viruses

**Figure 2:**
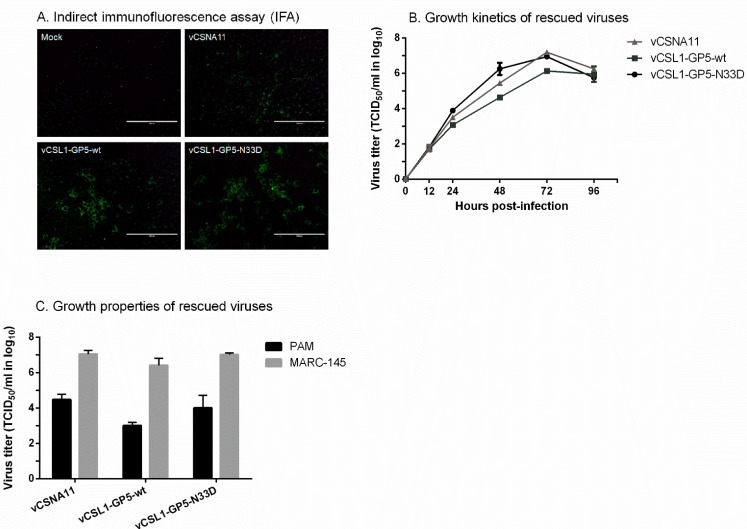
Characterization of mutant viruses. The in vitro growth characteristics were compared among vCSNA11, vCSL1-GP5-wt, and vCSL1-GP5-N33D. (**A**) Indirect immunofluorescence assay (IFA) was performed in MARC-145 cells to confirm the rescue of viruses. Bar, 200 μm. (**B**) The MARC-145 cells were infected at an MOI of 0.1 to compare the growth kinetics of rescued viruses. (**C**) Growth properties of rescued viruses were compared by titration in MARC-145 cells and PAMs.

A PRRSV field isolate KU-PRRSV-2020-002 was isolated and grew to high titers in PAMs but failed to grow in MARC-145 cells. According to a previous study, chimeric viruses containing gene regions of a MARC-145-unadaptive strain were not rescued or displayed retarded growth [19]. To attain a mutant virus with fine replicative fitness, the GP5 ectodomain region (93 bp) was selected for substitution of the gene regions instead of the whole structural protein-coding region (3.3 kb) of KU-PRRSV-2020-002 (Figure 1B). The GP5 ectodomain region includes three sites (amino acid positions 32–34, 38–39, and 57–59) related to cross-neutralization among PRRSV strains [20]. Therefore, the degree of cross-neutralization can be significantly increased by changing these three sites of mutant viruses into homologous amino acids with KU-PRRSV-2020-002. To further induce high levels of neutralizing antibodies, the hypo-glycosylated mutant virus was generated by amino acid substitution of GP5 N33 with D (Figure 1B). Previous studies reported that the hypo-glycosylated virus exhibits enhanced immunogenicity because the neutralizing epitope is exposed by deglycosylation [21,22]. Rescue of the mutant viruses, vCSL1-GP5-wt and vCSL1-GP5-N33D, was confirmed by IFA in MARC-145 cells (Figure 2A).

### 3.2. Characterization of Mutant Viruses

Growth curve results show that the growth kinetics of the two mutant viruses (vCSL1-GP5-wt and vCSL1-GP5-N33D) were comparable to those of the parental virus (vCSNA11) (Figure 2B). The parental and mutant viruses reached peak titers at 72 h p.i., although vCSL1-GP5-wt showed slightly lower peak titers than vCSNA11 and vCSL1-GP5-N33D.

The rescued viruses were able to grow in PAMs, which are the primary target cells for PRRSV in vivo. The viral titers in PAM cells were 400- to 2500-fold lower than those in MARC-145 cells (Figure 2C).

### 3.3. SVN Antibody Production upon Inoculation of Inactivated Vaccine of the Mutant Virus

**Figure 3:**
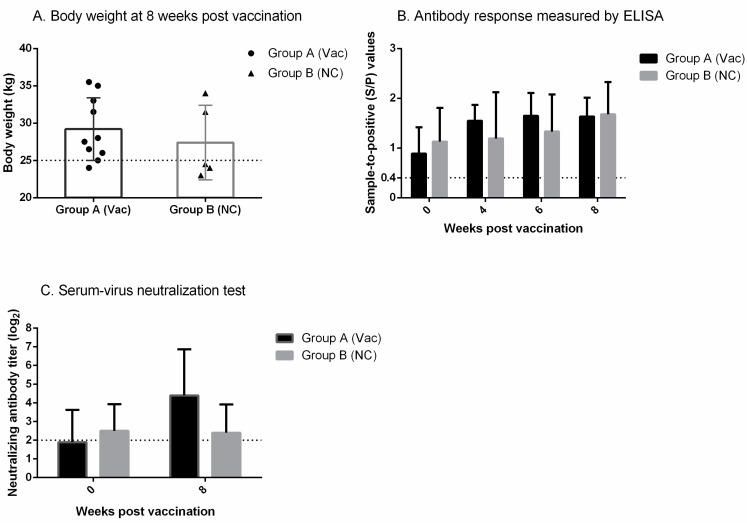
Results of the animal experiment: vaccine performance under field conditions. A farrow-to-finisher farm infected with PRRSV-2, KU-PRRSV-2020-002, was selected for the animal experiment. A total of 15 three-week-old piglets were randomly divided into groups A and B, which consisted of 10 and 5 pigs, respectively. Group A was immunized intramuscularly (IM) with the vCSL1-GP5-N33D inactivated vaccine (10^7.0^ TCID_50_/1 mL/dose), while group B was injected IM with PBS as negative control (NC). (**A**) Body weight at 8 weeks post-vaccination. The 25 kg was represented by the horizontal dotted line. (**B**) Antibody response measured by ELISA. The horizontal dotted line indicates the cutoff value of the test. (**C**) Neutralizing antibody titer measured against vCSL1-GP5-N33D. The horizontal dotted line indicates the cutoff value of the test.

In regard to the clinical signs, both groups (group A and B) remained normal throughout the study. The body weights of the vaccinated group (group A) at 8 wpv were relatively higher than those of the negative control (group B) (29.2 kg ± 4.2 vs. 27.4 kg ± 5.0), but the differences were not significant (*p* > 0.05) (Figure 3A, Supplementary Table S1). In the vaccinated group, 90% of pigs exhibited body weights of ≥25 kg, while only 40% of pigs in the negative control weighed ≥25 kg at 8 wpv (Table 1).

Sera of the vaccinated group and negative control collected at 0 wpv tested positive by ELISA (Figure 3B, Supplementary Table S1). At 4, 6, and 8 wpv, high sample-to-positive (S/P) values (>1.00) were detected in both groups, and the S/P values were comparable between the two groups.

Based on the SVN test results, the vaccinated group induced approximately 2-fold higher SVN antibody titers, i.e., 4.40 (log_2_) ± 2.46 vs. 2.40 (log_2_) ± 1.52, than the negative control at 8 wpv (Figure 3C, Supplementary Table S1).

The proportion of pigs displaying SVN antibody titers of ≥5 (log_2_) at 8 wpv was significantly (*p* ≤ 0.05) higher in the vaccinated group than in the negative control (Table 1).

## 4. Discussion

The GP5 of PRRSV is a major envelope glycoprotein and thus acts as the main target for neutralizing antibodies [21]. The epitope in the GP5 ectodomain is reported to be the primary neutralization epitope of PRRSV [23]. In the GP5 ectodomain, there are three critical sites (amino acid positions 32–34, 38–39, and 57–59) that are known to determine the susceptibility to neutralizing antibodies [20]. According to a previous study, the titers of neutralizing antibodies were 0 (log_2_) when one critical site was identical between the two PRRSV strains but raised to 3 (log_2_) by point mutation of another critical site into homologous amino acid [20]. In this study, the entire GP5 ectodomain, including all three critical sites, was replaced with that of the lineage-1 PRRSV-2 strain (i.e., KU-PRRSV-2020-002) to elicit neutralizing antibodies in an enhanced manner.

The neutralizing antibody responses against PRRSV are generally slow and weak, which is related to the glycan-moieties present on the GP5 ectodomain [24]. N34 (it could be N33 or N35 in some strains), N44, and N51 are used for N-linked glycosylation to generate fully glycosylated wild-type PRRSV GP5 [21]. These N-glycans mask the nearby major neutralization epitope and save the virus from neutralization [24]. Accordingly, the loss of glycan residues exposes the neutralization epitope and enhances the immunogenicity of the virus [21]. A previous study has shown that a mutant PRRSV carrying a single deglycosylation mutation at GP5 N44 induced 2-fold higher levels of neutralizing antibodies compared to the wild-type virus [22]. Likewise, mutant PRRSVs with single or double deglycosylation mutations at GP5 N34 and N55 produced 2- to 3-fold higher neutralizing antibody titers than the wild-type virus [21]. In this research, a single deglycosylation mutation at GP5 N33 was applied to the chimeric virus of KU-PRRSV-2020-002 to increase the production of neutralizing antibodies. The hypo-glycosylated chimeric virus (i.e., vCSL1-GP5-N33D) was then produced as an inactivated vaccine that can be safely applied to a swine herd.

In previous studies, PRRSV inactivated vaccines have shown questionable efficacy, especially in PRRS-free herds [4]. The immunogenicity of inactivated vaccines was so low that ELISA antibody responses were not detected for as long as 10 wpv, and the vaccines did not evoke any detectable level of protective immunity after challenges [25,26]. However, improved immunogenicity was observed in inactivated vaccines with hypo-glycosylated GP5 [16,27]. FL12/GP5DM is a mutant PRRSV with double deglycosylation mutations at GP5 N34 and N51, and pigs vaccinated with FL12/GP5DM inactivated vaccine displayed neutralizing antibody titers of 3 (log_2_) at 6 wpv [16]. Furthermore, the FL12/GP5DM inactivated vaccine conferred protection against homologous challenges, as demonstrated by significantly lower levels of viremia and microscopic lung lesion scores compared to the unvaccinated group [16]. Similarly, K418/GP5DM, a mutant PRRSV with double deglycosylation mutations at GP5 N33 and N51, was applied as an inactivated vaccine to a PPRS-positive herd and elicited high neutralizing antibody titers of 4.6 (log_2_) at 7 wpv [27]. Consistent with the results of previous studies, an inactivated vaccine of vCSL1-GP5-N33D induced high SVN antibody titers of 4.4 (log_2_) at 8 wpv in a PRRS-positive herd.

The production of neutralizing antibodies is usually limited in PRRSV infection, and PRRSV-infected pigs can clear the virus in the absence of neutralizing antibodies [28]. Nevertheless, it is generally accepted that a sufficient amount of neutralizing antibodies can protect pigs from PRRSV infection [29]. The amount of neutralizing antibodies needed for protection is higher in piglets than in sows, presumably because of the age dependence of the PRRSV infection [30]. In piglets, a neutralizing antibody titer of ≥3 (log_2_) can block the viremia, and a titer of ≥5 (log_2_) provides complete protection against PRRSV infections [30]. The present study attained a mean neutralizing antibody titer of ≥3 (log_2_), and 60% of pigs had an antibody titer of ≥5 (log_2_). The result implies vCSL1-GP5-N33D inactivated vaccine is protective against PRRSV infections through high levels of neutralizing antibodies.

In the current study, there were no significant differences in the body weights between the vaccinated group and the negative control. However, the mean body weights were relatively higher in the vaccinated group than in the negative control, so the results were interpreted as an improvement in the growth performance of pigs. Previous studies have shown that conventional administration of the inactivated vaccine to PPRS-positive herds can significantly improve the health status of pigs, which agrees with the results of the present study [31,32].

The efficacy of a PRRSV inactivated vaccine also depends on the viral inactivation method and the adjuvant [33,34]. In a previous study, PRRSV was inactivated by various procedures and examined for the intactness of the viral entry-associated domains [33]. Of the inactivation methods, including formaldehyde, glutaraldehyde, ultraviolet radiation, gamma irradiation, and BEI, the latter could preserve the entry-associated domains and was regarded as safe from photoproducts or free radicals. In this study, high neutralizing antibody titers were observed in pigs vaccinated with BEI-inactivated virus, which indicates that entry-associated domains as well as neutralizing epitopes were conserved during inactivation. Meanwhile, diverse adjuvants, including Incomplete Freund’s Adjuvant, 16% aluminum hydroxide colloidal gel, and oil-in-water (o/w) diluent, were assessed for immunogenicity in combination with PRRSV antigens [34]. The choice of adjuvants influenced the immunogenicity of neutralizing epitopes, and o/w adjuvant induced the strongest neutralizing antibody responses. The present study used Montanide IMS1313 VG adjuvant based on previous publications [16,27] and confirmed that the vaccine was appropriately immunogenic and effective.

## 5. Conclusions

To the best of our knowledge, this is the first study to develop a vaccine against Korean lineage-1 PRRSV. In summary, a vaccine candidate against lineage-1 PRRSV was successfully generated using reverse genetics technology and named vCSL1-GP5-N33D. vCSL1-GP5-N33D was administered to a PRRS-positive farm in the form of an inactivated vaccine and induced high levels of SVN antibody. Since this study is not a vaccination/challenge experiment, the protective efficacy of the vaccine candidate needs to be further evaluated under experimental conditions. Nevertheless, the vCSL1-GP5-N33D inactivated vaccine showed good performance in a PRRS-positive herd and proved to be a promising vaccine candidate. The inactivated vaccine of vCSL1-GP5-N33D is expected to contribute to PRRS control by imposing a therapeutic effect in PRRS-endemic Korean farms through high SVN antibody titers. Furthermore, the methodology used in the generation of vCSL1-GP5-N33D (i.e., the platform of chimeric hypo-glycosylated virus) can be applied to the production of new vaccines against emerging PRRSV variants.

## Figures and Tables

**Table 1:** Results of the animal experiment: body weight and serum virus neutralization (SVN) antibody titer of the two groups at 8 weeks post-vaccination (wpv). The proportion of pigs showing body weight of <25 kg or ≥25 kg and pigs with an SVN antibody titer of <5 (log_2_) or ≥5 (log_2_) of the vaccinated group (group A, Vac) and negative control (group B, NC) at 8 wpv.

		Group		Two-Tailed *p* Value
		A (Vac)	B (NC)	
Body weight at 8 wpv	<25 kg	1/10	3/5	0.077
	≥25 kg	9/10	2/5	
SVN antibody titer at 8 wpv	<5 (log_2_)	4/10	5/5	0.044
	≥5 (log_2_)	6/10	0/5	

## Data Availability

Data available from the corresponding author on reasonable request.

## Supplementary Materials

The following supporting information can be downloaded at: https://www.mdpi.com/article/10.3390/vetsci9040165/s1, Table S1: Results of the animal experiment.
