# Supplementary material for: Development of a Chimeric Porcine Reproductive and Respiratory Syndrome Virus (PRRSV)-2 Vaccine Candidate Expressing Hypo-Glycosylated Glycoprotein-5 Ectodomain of Korean Lineage-1 Strain
Source: Vet Sci. 2022 Mar 29;9(4):165. doi: 10.3390/vetsci9040165 (PMC9028511; doi:10.3390/vetsci9040165)
Supplement: Supplementary file 1 [file vetsci-09-00165-s001.zip › vetsci-1630112-supplementary.pdf]

Supplementary Table S1. Results of the animal experiment. Body weight (BW), sample-to-positive (S/P) values, and serum virus neutralization (SVN) antibody titer of the vaccinated group (group A, Vac) and negative control (group B, NC) at different weeks post-vaccination (wpv). The cutoff values of the ELISA and SVN test are 0.40 and 2.00, respectively.

		**BW**	**S/P values**				**SVN antibody titer**	
**Groups**	**Pigs no.**	**8 wpv**	**0 wpv**	**4 wpv**	**6 wpv**	**8 wpv**	**0 wpv**	**8 wpv**
**Group A (Vac)**	1	35.0	0.86	1.32	1.03	1.98	2.00	6.00
	2	27.5	1.76	0.99	0.95	1.46	4.00	5.00
	3	33.0	1.39	1.34	2.23	1.57	3.00	8.50
	4	31.5	0.36	2.13	2.00	1.72	0.00	5.75
	5	24.0	1.13	1.76	1.88	2.04	4.00	5.00
	6	35.5	1.47	1.69	2.00	1.02	3.00	6.00
	7	25.0	0.48	1.37	1.34	1.42	0.00	2.00
	8	26.0	0.67	1.82	1.47	1.97	3.00	0.00
	9	26.5	0.11	1.54	1.49	2.27	0.00	2.75
	10	28.0	0.66	1.53	2.13	2.02	0.00	3.00
**Group B (NC)**	1	31.5	1.96	1.43	1.80	1.38	3.00	2.00
	2	34.0	0.30	0.01	0.02	0.01	0.00	0.00
	3	24.5	0.75	2.22	2.13	1.41	4.00	3.00
	4	24.0	1.42	1.62	1.95	1.95	3.00	4.00
	5	23.0	1.94	1.81	1.01	1.87	2.50	3.00
